# An 8-ferroptosis-related genes signature from Bronchoalveolar Lavage Fluid for prognosis in patients with idiopathic pulmonary fibrosis

**DOI:** 10.1186/s12890-021-01799-7

**Published:** 2022-01-05

**Authors:** Yaowu He, Yu Shang, Yupeng Li, Menghan Wang, Dongping Yu, Yi Yang, Shangwei Ning, Hong Chen

**Affiliations:** 1 Department of Respiratory and Critical Care Medicine, Second Affiliated Hospital of Harbin Medical University, Harbin, 150086 China; 2 Department of Respiration, The First Hospital of Harbin, Harbin, 150010 China; 3 College of Bioinformatics Science and Technology, Harbin Medical University, Harbin, 150086 China

**Keywords:** Idiopathic pulmonary fibrosis, Gene signature, Ferroptosis, Prognosis, Inflammation, Immune response

## Abstract

**Background:**

With the rapid advances of genetic and genomic technologies, the pathophysiological mechanisms of idiopathic pulmonary fibrosis (IPF) were gradually becoming clear, however, the prognosis of IPF was still poor. This study aimed to systematically explore the ferroptosis-related genes model associated with prognosis in IPF patients.

**Methods:**

Datasets were collected from the Gene Expression Omnibus (GEO). The least absolute shrinkage and selection operator (LASSO) Cox regression analysis was applied to create a multi-gene predicted model from patients with IPF in the Freiburg cohort of the GSE70866 dataset. The Siena cohort and the Leuven cohort were used for validation.

**Results:**

Nineteen differentially expressed genes (DEGs) between the patients with IPF and control were associated with poor prognosis based on the univariate Cox regression analysis (all *P* < 0.05). According to the median value of the risk score derived from an 8-ferroptosis-related genes signature, the three cohorts’ patients were stratified into two risk groups. Prognosis of high-risk group (high risk score) was significantly poorer compared with low-risk group in the three cohorts. According to multivariate Cox regression analyses, the risk score was an independently predictor for poor prognosis in the three cohorts. Receiver operating characteristic (ROC) curve analysis and decision curve analysis (DCA) confirmed the signature's predictive value in the three cohorts. According to functional analysis, inflammation- and immune-related pathways and biological process could participate in the progression of IPF.

**Conclusions:**

These results imply that the 8-ferroptosis-related genes signature in the bronchoalveolar lavage samples might be an effective model to predict the poor prognosis of IPF.

**Supplementary Information:**

The online version contains supplementary material available at 10.1186/s12890-021-01799-7.

## Introduction

Characterized by fibrosis or structural deformations, idiopathic pulmonary fibrosis (IPF) is a chronic and progressive interstitial lung disease of unknown etiology [1, 2]. In the United States, incidence of IPF in people aged 18–64 years was 6.1 new cases per 100,000 person-years [3]. Currently, pirfenidone and nintedanib have been approved by the United States Food and Drug Administration to treat patients with IPF [4, 5]. However, the prognosis of IPF is still poor, the median survival time is usually 2–3 years after diagnosis [6, 7], and the 5-year survival rate is less than 40% [8]. Therefore, it is very important to explore the novel prognostic models associated with prognosis in patients with IPF.

As a kind of special biological sample, bronchoalveolar lavage fluid (BALF) contains the accumulation of extravasated inflammatory cells and cytokines reflecting the microenvironment around the alveoli, and studies have verified that changes in the alveolar microenvironment are correlated with the progression of IPF [9]. A study has constructed a 4-genes signature of bronchoalveolar lavage cells, which may be an effective prognostic tool to deliver more personalized treatment decisions for patients with IPF [10]. Ferroptosis is a newly described form of caspase-independent regulated cell death (RCD) characterized by cellular accumulation of reactive oxygen species (ROS) driven through iron-dependent lipid [11, 12]. Studies have found that ferroptosis was associated with the fibrosis of many organs such as liver, heart, and lung, and the ROS accumulation and oxidative stress may be the primary inducer of ferroptosis in this process [13–17]. In addition, iron overload may cause lung fibrosis according to increased lipid peroxidation and decreased glutathione peroxidase 4 (GPX4) activity in lung tissues [18]. However, whether the ferroptosis-related genes are associated with prognosis of patients with IPF is unclear. Furthermore, the systematic exploration of the prognostic signature based on ferroptosis-related genes between the patients with IPF and subjects in the control group is also absent.

Therefore, according to the dataset from the Gene Expression Omnibus (GEO), the study aimed to systematically explore the prognostic value of an 8-ferroptosis-related genes signature in patients with IPF. Finally, we explored the possible mechanisms based on functional enrichment analysis.

## Materials and methods

### Acquisition of data

**Fig. 1:**
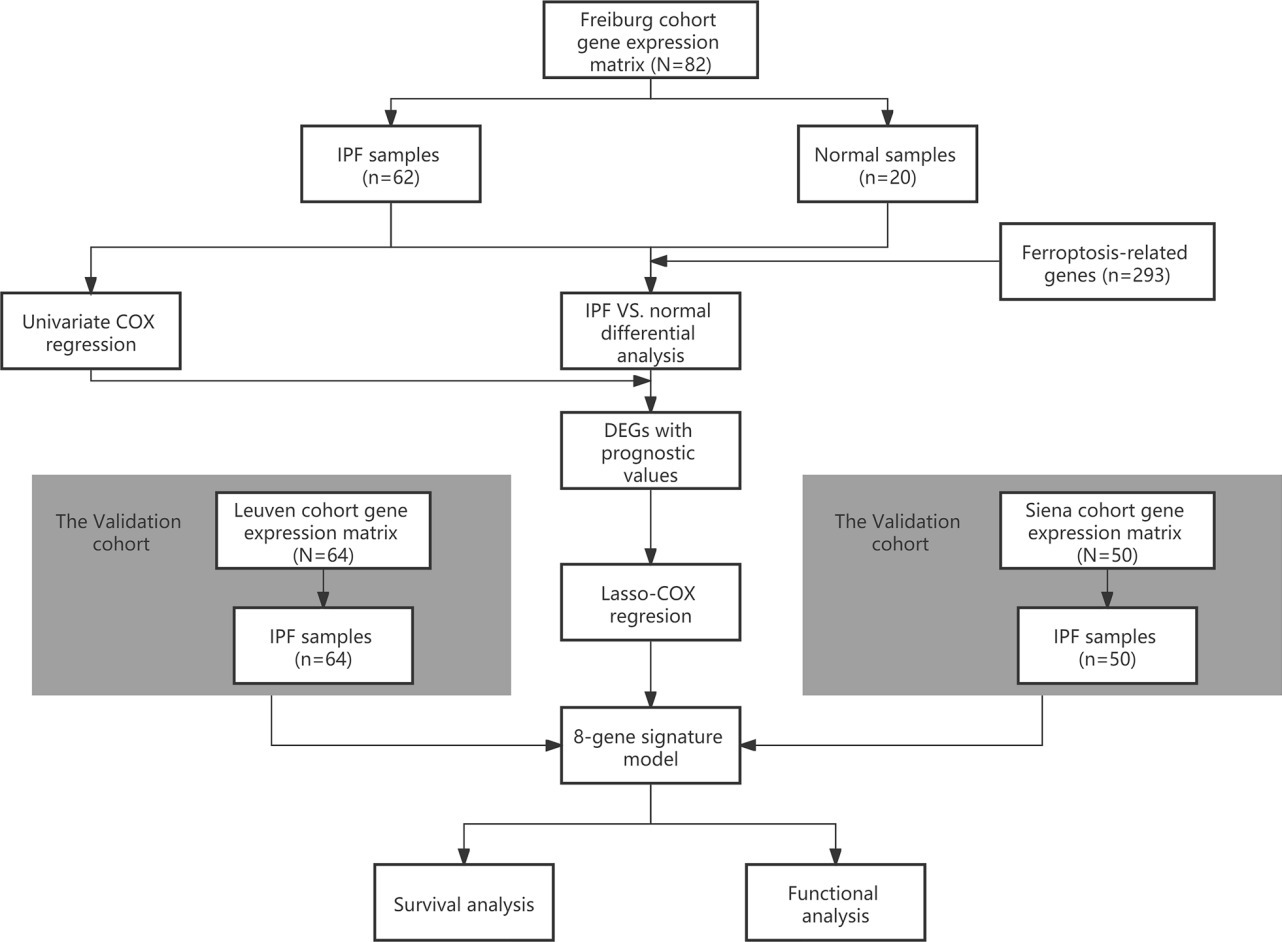
Workflow of this study

The workflow of this study was shown in Fig. 1. On the GEO database (http://www.ncbi.nlm.nih.gov/geo/), we selected datasets must meet the following items: (1) the detected samples came from the bronchoalveolar lavage (BAL) of patients with IPF or control group; (2) raw data or a gene expression matrix should be provided; (3) the dataset must contain information regarding the prognostic status. Finally, one dataset, GSE70866 was identified (platform: GPL14550 and GPL17077). IPF diagnosis in this dataset was established by a multidisciplinary board at each institution based on the American Thoracic Society/European Respiratory Society criteria [19, 20] and was confirmed to be consistent with guidelines published in 2011 [2]. Approval of the Ethics Committee was not required because the information of patients was obtained from the GEO.

Ferroptosis-related genes (FRGs) were acquired from GeneCards database (https://www.genecards.org/) by searching the terms “ferroptosis” and PubMed by searching the terms “Ferroptosis [MeSH] OR Ferroptosis* [tiab]”. Consequently, 103 FRGs and 190 FRGs were respectively obtained from GeneCards and PubMed in the study (Additional file 7: Table S1).

### Construction and validation of a prognostic ferroptosis-related gene signature

This study included a discovery cohort consisted of 20 normal people and 62 patients from Freiburg, Germany, and two independent validation cohorts: Siena, Italy (50 patients) and Leuven, Belgium (64 patients, Table 1). The differentially expressed genes (DEGs) between patients with IPF and controls were identified by R package "limma" with a false discovery rate (FDR) < 0.05 in the Freiburg cohort [21]. Cox regression analysis was performed for prognostic value of the ferroptosis-related genes according to R package “survival” (https://github.com/therneau/survival, v.3.2–7) or SPSS Statistics 23 (IBM SPSS). Heatmap was constructed according to R packages “pheatmap” (v1.0.12). An interaction network for the ferroptosis-related DEGs with significant prognostic value was generated by the STRING database (version 11.0) [22] and visualized by Cytoscape (a software platform for visualizing complex networks, version v3.6.1). In order to minimize the risk of overfitting, a prognostic model was constructed by LASSO-penalized (least absolute shrinkage and selection operator) Cox regression analysis according to the R package "glmnet" (v.4.1-1) [23, 24]. The independent variable in the Cox regression was the expression matrix of candidate ferroptosis-related DEGs with significant prognostic value, and the response variables were survival status of patients in the Freiburg cohort. Following the minimum criteria, penalty parameter (λ) for the model was determined by ten-fold cross-validation (the value of λ corresponding to the lowest partial likelihood deviance). The risk scores of the patients were calculated based on each gene’s expression level and its corresponding regression coefficient as follows: score = sum (each gene’s expression × corresponding coefficient). The patients were stratified into high-risk and low-risk groups according to the median value of the risk score. Based on the expression of genes in the predicted model, principal component analysis (PCA) was performed in the GraphPad Prism 9. Besides, t-distributed stochastic neighbor embedding (t-SNE) were carried out to seek the distribution of different groups using the "Rtsne" R package (v.0.15). The optimal cut-off expression value of each gene of the model was determined for the survival analysis by the "surv_cutpoint" function of the R package "survminer" (https://cran.r-project.org/web/packages/survminer/index.html, v.0.4.9). The time‐dependent ROC curve was constructed to evaluate the predictive value of the risk score according to the R package "survivalROC" (https://CRAN.R-project.org/package=survivalROC,v.1.0.3).

**Table 1:** Clinical characteristics of the patients with IPF used in this study

Characteristics	Freiburg cohort	Siena cohort	Leuven cohort
No. of patients	62	50	64
Age (median, range)	69 (63–75)	69 ± 11	68 ± 9
*Gender (%)*			
Female	9 (14.5)	10 (20.0)	13 (20.3)
male	53 (85.5)	40 (80.0)	51 (79.7)
*GAP*			
Stage I	17 (27.4)	14 (28.0)	25 (39.1)
Stage II	32 (51.6)	20 (40.0)	31 (48.4)
Stage III	13 (21.0)	16 (32.0)	8 (12.5)
*Survival status*			
Time to death (months)	18 (6–32)	14 ± 9	12 (7–24)
Censored	17 (27.4)	19 (38.0)	40 (62.5)

Values are presented as median (IQR), mean ± SD or n (%)

*GAP* gender, age and physiologic variables, *IQR* interquartile range, *SD* standard deviation

Decision curve analysis (DCA) was performed to evaluate whether the prognostic model could help improve clinical decision-making. The y-axis represents the net benefit, and the x-axis indicates the probability of the threshold value. By using “stdca.R” (http://www.decisioncurveanalysis.org/) [25], DCA was used to determine the clinical usefulness of the signature.

### Functional enrichment analysis

Based on the DEGs (|log2FC|> 1, FDR < 0.05) between the high-risk and low-risk groups, Gene Ontology (GO) and Kyoto Encyclopedia of Genes and Genomes (KEGG) analyses were conducted by the R package "clusterProfiler" (v.3.18.1) [26]. *P* values were adjusted with the Benjamini & Hochberg (BH) correction. The infiltrating score of 19 immune cells and the activity of 15 immune-related pathways (Additional file 8: Table S2) [27] were calculated with single-sample gene set enrichment analysis (ssGSEA) using the R package "gsva" (v.1.38.2) [28].

### Statistical analysis

R software (Version 4.0.3) and SPSS Statistics 23 (IBM SPSS) was used for statistical analysis. When the data were normally distributed, means for continuous variables were compared according to independent group t tests [described as mean ± SD (standard deviation)]; otherwise, the Mann–Whitney test was used (data were described as median [interquartile range [IQR])]. Categorical variables were described as number (%) and were compared by Chi-square test or the Fisher exact test. Kaplan–Meier analysis with the log-rank test was used to compare the survival between different groups. Univariate and multivariate Cox regression was used to estimate the hazard ratio (HR) to identify predictors of mortality. Some statistical analyses were visualized by GraphPad Prism 9. Bilateral test (the test level α = 0.05) was used.

## Results

### Identification of prognostic ferroptosis-related DEGs in the Freiburg cohort

**Fig. 2:**
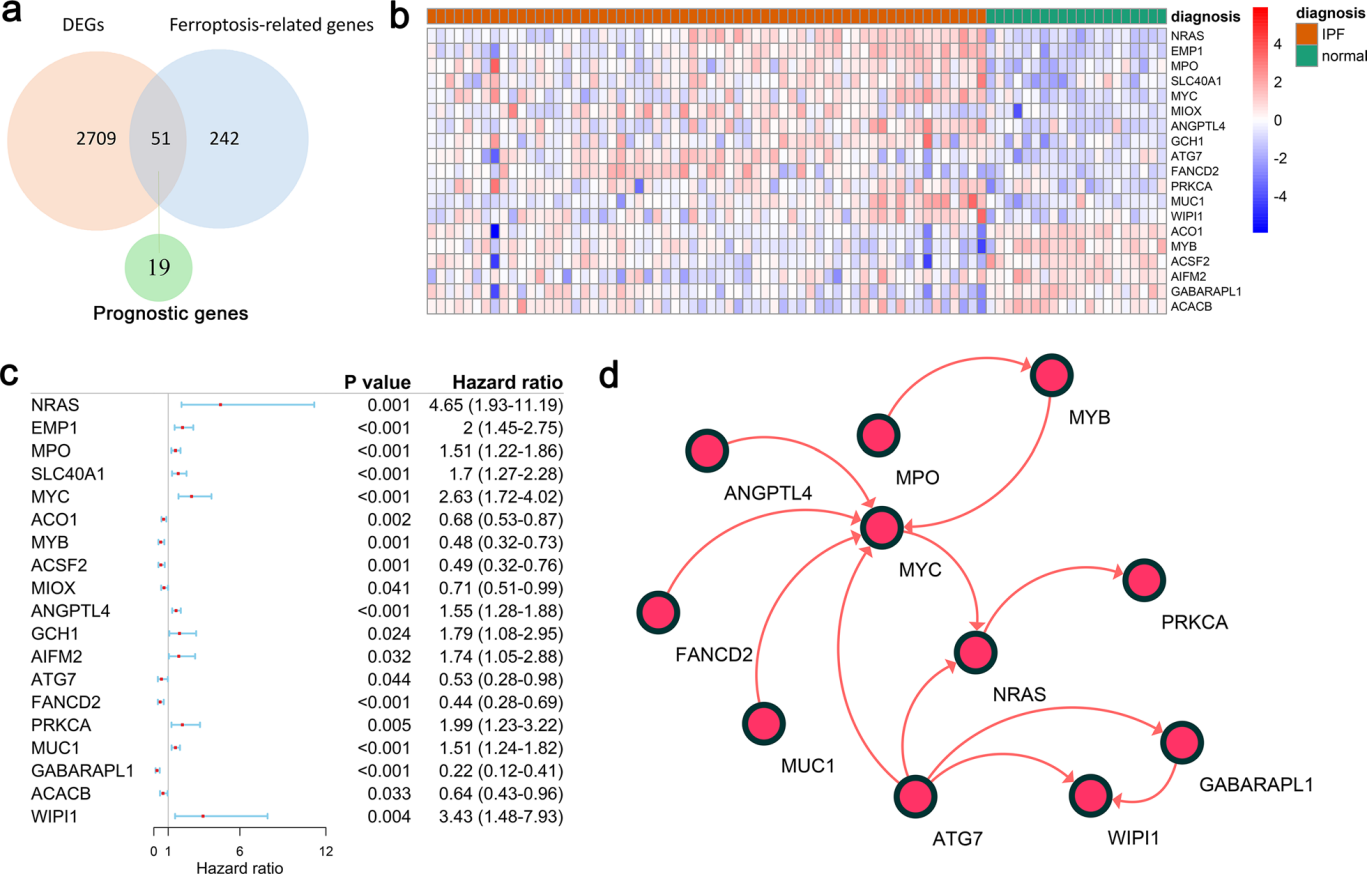
Identification of prognostic genes in the Freiburg cohort. (**a**) The intersection of genes between the DEGs and ferroptosis-related genes. (**b**) Heatmap plot of 19 prognostic DEGs. Red represents expression level of genes were up-regulated, blue represents expression level of genes were down-regulated. A relative color scheme uses the minimum and maximum values in each row to convert values to colors. (**c**) Forest plots of the univariate Cox regression analysis of the 19 prognostic DEGs. (**d**) A protein–protein interaction network for the 19 prognostic DEGs, 8 genes were excluded due to no connection with other genes

Of the 293 ferroptosis-related genes, 51 (17.4%) were differentially expressed between patients with IPF and controls, and 19 of them were correlated with prognosis according to the univariate Cox regression analysis (Fig. 2a–c). The interaction network among the 19 genes was showed in Fig. 2d.

### Construction of a prognostic model in the Freiburg cohort

**Fig. 3:**
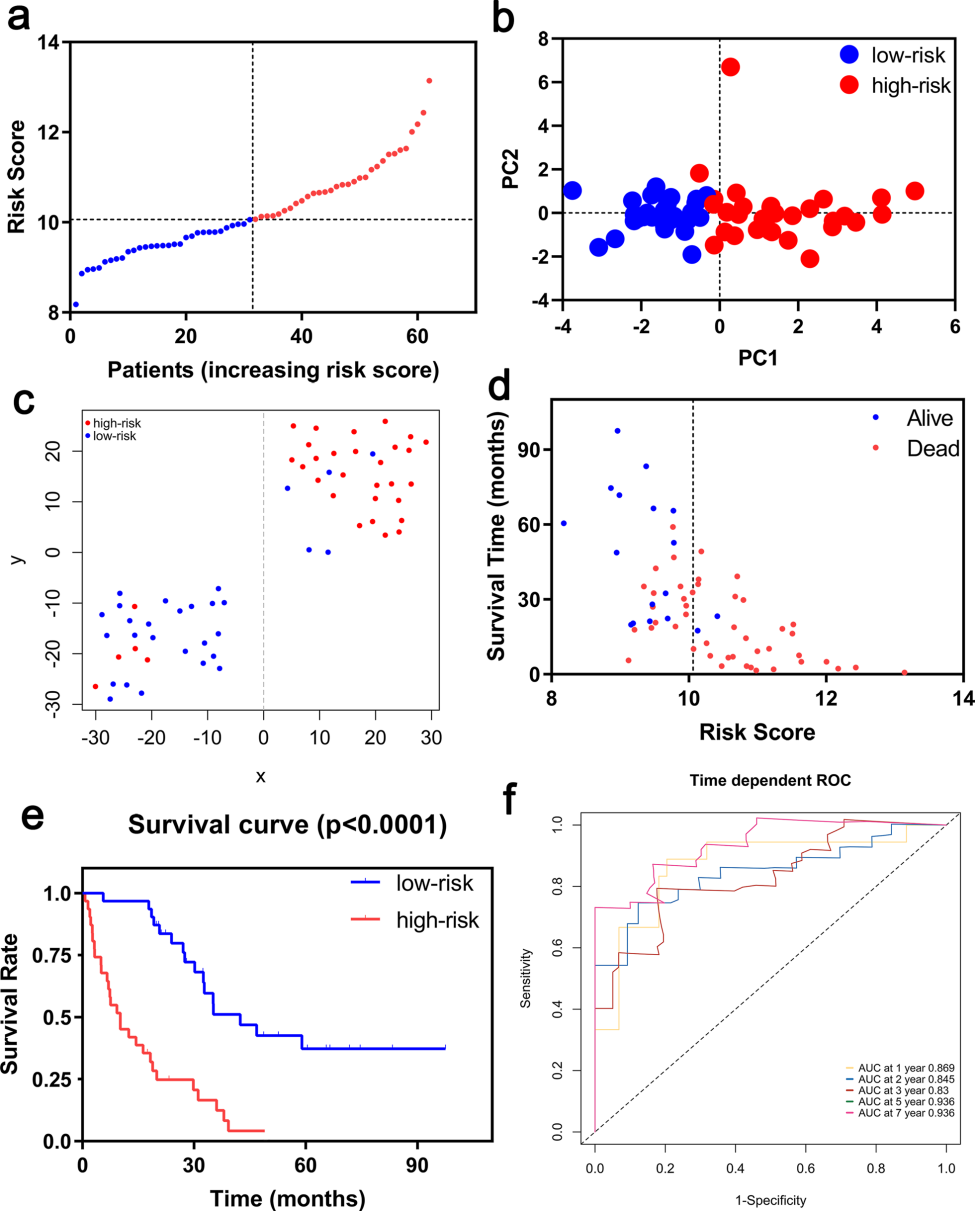
Prognostic analysis of the 8-gene signature model in the Freiburg cohort. (**a**) The distribution and median value of the risk scores. (**b**) PCA plot. (**c**) t-SNE analysis. (**d**) The distributions of survival status, survival time and risk score. (**e**) Kaplan–Meier curves for the survival between high-risk and low-risk patients. (**f**) Time-dependent ROC curves for the predictive value of the risk score

A prognostic model was established by LASSO Cox regression analysis using the expression profile of the 19 genes mentioned above. An 8-ferroptosis-related genes signature was constructed based on the optimal value of λ (Additional file 1: Figure S1), and the survival analyses of the 8 genes according to the optimal cut-off expression value of each gene were showed in the Additional file 2: Figure S2. The risk score was calculated as follows: 0.447056157 * expression level of NRAS + 0.008853087 * expression level of EMP1 + 0.283483044 * expression level of SLC40A1 + 0.308932641 * expression level of MYC + 0.191156392 * expression level of ANGPTL4 + 0.312166561 * expression level of PRKCA + 0.072692152 * expression level of MUC1—0.200150039 * expression level of GABARAPL1. According to the median cut-off value, the patients were stratified into a high-risk group (n = 31) and a low-risk group (n = 31) (Fig. 3a). The risk score was significantly positively correlated with GAP score and mortality in the Freiburg cohort (Table 2). PCA and t-SNE analysis demonstrated the patients in different risk groups were distributed in two directions (Fig. 3b, c). Patients in the high-risk group had a higher probability of death earlier than those in the low-risk group (Fig. 3d, e). Time-dependent ROC curves was used to evaluated the predictive performance of the risk score for mortality, and the area under the curve (AUC) reached 0.869 at 1 year, 0.845 at 2 years, 0.83 at 3 years and 0.936 at 5, 7 years (Fig. 3f).

**Table 2:** Baseline characteristics of the patients in different risk groups

Characteristics	Freiburg cohort			Siena cohort			Leuven cohort		
	High risk	Low risk	*P* value	High risk	Low risk	*P* valve	High risk	Low risk	*P* value
*Age, years*	65.74 ± 10.06	69.06 ± 7.82	0.262	70.08 ± 11.34	67.28 ± 11.13	0.383	68.53 ± 7.80	67.97 ± 9.35	0.795
≥ 60	22 (71.0)	27 (87.1)	0.119	20 (80.0)	22 (88.0)	0.702	29 (90.6)	27 (84.4)	0.708
< 60	9 (29.0)	4 (12.9)		5 (20.0)	3 (12.0)		3 (9.4)	5 (15.6)	
*Gender*			1.000			0.480			0.120
Female	5 (16.1)	4 (12.9)		4 (16.0)	6 (24.0)		4 (12.5)	9 (28.1)	
Male	26 (83.9)	27 (87.1)		21 (84.0)	19 (76.0)		28 (87.5)	23 (71.9)	
*GAP*			0.027			0.051			0.080
Stage I	4 (12.9)	13 (41.9)		5 (20.0)	9 (36.0)		8 (25.0)	17 (53.1)	
Stage II	18 (58.1)	14 (45.2)		8 (32.0)	12 (48.0)		19 (59.4)	12 (37.5)	
Stage III	9 (29.0)	4 (12.9)		12 (48.0)	4 (16.0)		5 (15.6)	3 (9.4)	
*Survival status*			< 0.001			0.001			0.002
Dead	29 (93.5)	16 (51.6)		21 (84.0)	10 (40.0)		18 (56.3)	6 (18.8)	
Censored	2 (6.5)	15 (48.4)		4 (16.0)	15 (60.0)		14 (43.7)	26 (81.2)	

Values are presented as mean ± SD or n (%)

*GAP* gender, age and physiologic variables, *SD* standard deviation

The decision curves of the prognostic model in the Freiburg cohort were shown in Additional file 3: Figure S3a. The black solid line indicates no intervention, and the net benefit is zero. The gray solid line represents the intervention, at which the net benefit is an oblique line with a negative slope. The red dotted line represents the realized profits of the 8-ferroptosis-related genes model. The decision curve showed that, at threshold values of 0.2–0.5, the greatest clinical benefit will be obtained from the prognostic model with 8-ferroptosis-related genes.

### Validation of the signature in the Siena and Leuven cohort

**Fig. 4:**
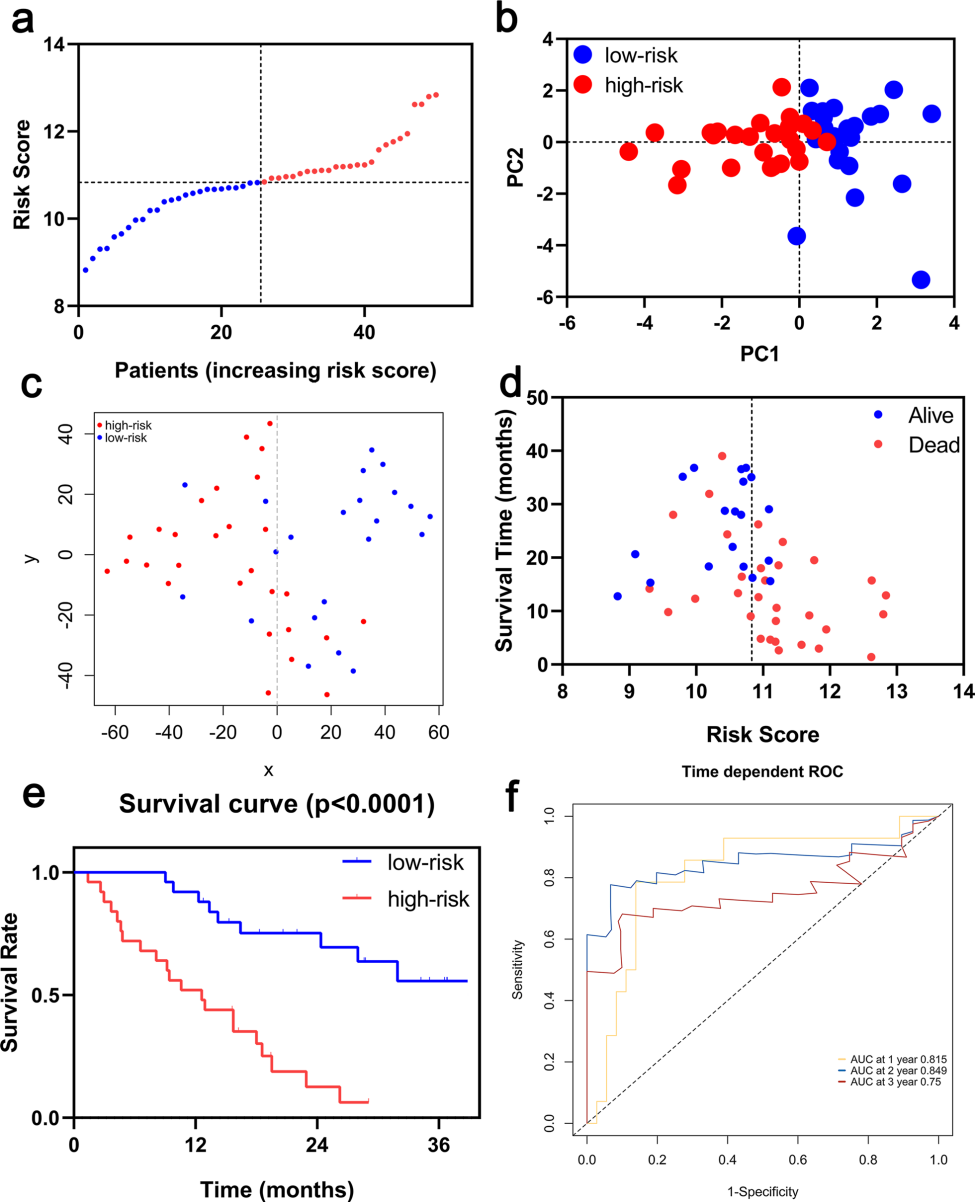
Prognostic analysis of the 8-gene signature model in the Siena cohort. (**a**) The distribution and median value of the risk scores. (**b**) PCA plot. (**c**) t-SNE analysis. (**d**) The distributions of survival status, survival time and risk score. (**e**) Kaplan–Meier curves for the survival between high-risk and low-risk patients. (**f**) Time-dependent ROC curves for the predictive value of the risk score

**Fig. 5:**
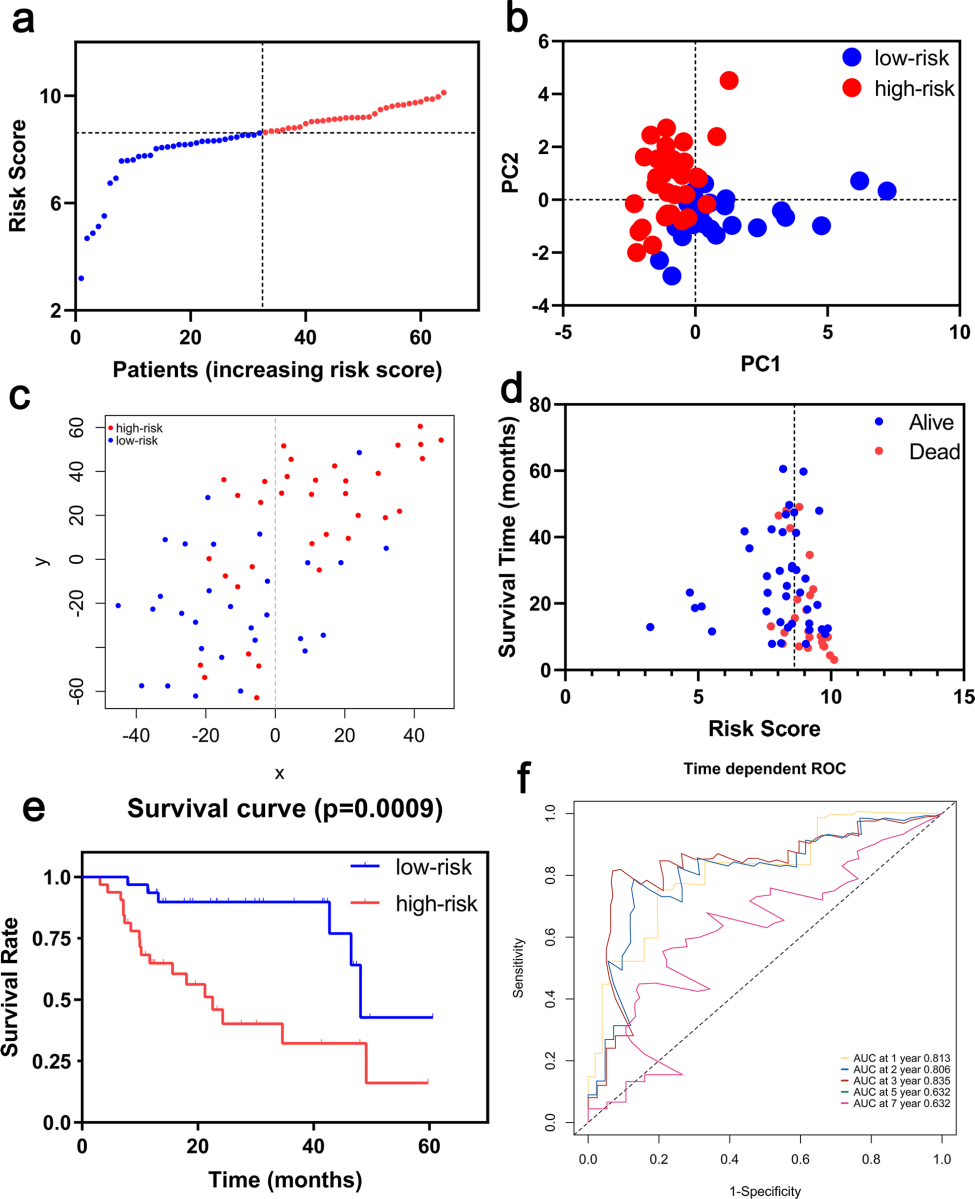
Prognostic analysis of the 8-gene signature model in the Leuven cohort. (**a**) The distribution and median value of the risk scores. (**b**) PCA plot. (**c**) t-SNE analysis. (**d**) The distributions of survival status, survival time and risk score. (**e**) Kaplan–Meier curves for the survival between high-risk and low-risk patients. (**f**) Time-dependent ROC curves for the predictive value of the risk score

Survival analyses of the 8 genes in the ferroptosis-related signature showed that these genes were associated with poor prognosis in the Siena cohort and the Leuven cohort (all P < 0.05, Additional files 4, 5: Figures S4, S5). To test the robustness of the signature constructed from the Freiburg cohort, the patients from the Siena and Leuven cohorts were also divided into high- or low-risk groups respectively according to the median value of risk score calculated with the same formula in the Freiburg cohort (Figs. 4a, 5a, respectively). The patients in the high-risk group were also associated with higher mortality in the Siena cohort and Leuven cohort, respectively (Table 2). PCA and t-SNE analysis demonstrated that patients in two subgroups were distributed in discrete directions (Figs. 4b, c, 5b, c). Consistently, high-risk patients were more likely to encounter death earlier and had higher mortality compared with low-risk patients (Figs. 4d, e, 5d, e). In addition, the AUC of the 8-genes signature was 0.815 at 1 year, 0.849 at 2 years, and 0.750 at 3 years in Siena cohort (Fig. 4f), and, the AUC of the 8-gene signature was 0.813 at 1 year, 0.806 at 2 years, 0.835 at 3 years and 0.632 at 5 years in Leuven cohort (Fig. 5f). Furthermore, The DCA showed that the risk score was more likely to have better clinical benefit than GAP (Additional file 3: Figure S3b–c).

### Independent prognostic value of the signature

**Fig. 6:**
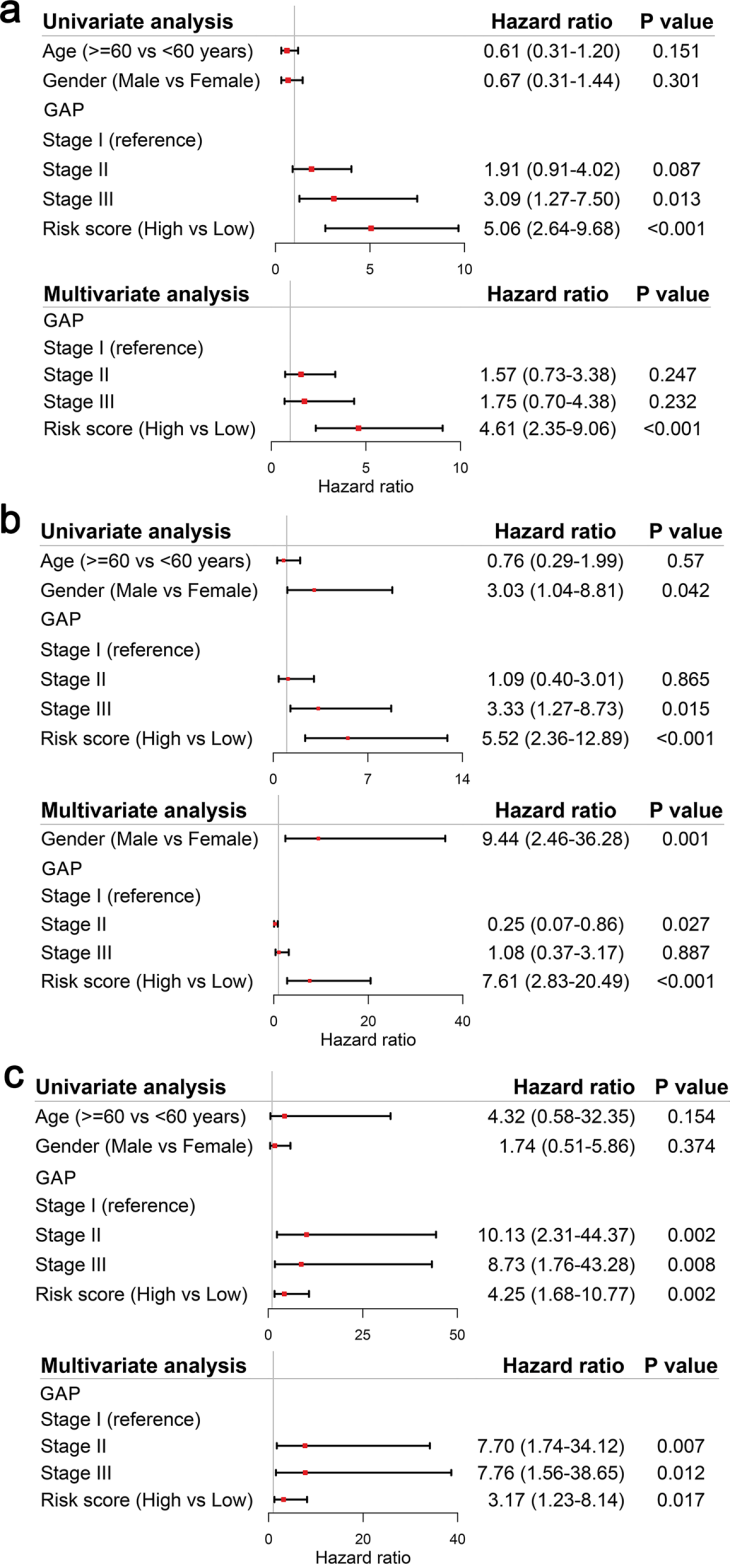
Results of the univariate and multivariate Cox regression analyses regarding mortality in the Freiburg cohort (**a**) and in the Siena validation cohort (**b**), and in the Leuven validation cohort (**c**)

In univariate Cox regression analyses, the risk score was significantly positively correlated with mortality in the Freiburg cohort, Siena cohort and Leuven cohort (HR = 5.06, 95% CI 2.64–9.68, P < 0.001; HR = 5.52, 95% CI 2.36–12.89, P < 0.001; HR = 4.25, 95% CI 1.68–10.77, P = 0.002, respectively, Fig. 6). After correcting for other confounding factors, the risk score was still an independent predictor for mortality in multivariate Cox regression analysis (Freiburg cohort: HR = 4.61, 95% CI 2.35–9.06, P < 0.001; Siena cohort: HR = 7.61, 95% CI 2.83–20.49, P < 0.001; Leuven cohort: HR = 3.17, 95% CI 1.23–8.14, *P* = 0.017; Fig. 6).

### Functional analyses in the Freiburg and the Siena cohort

**Fig. 7:**
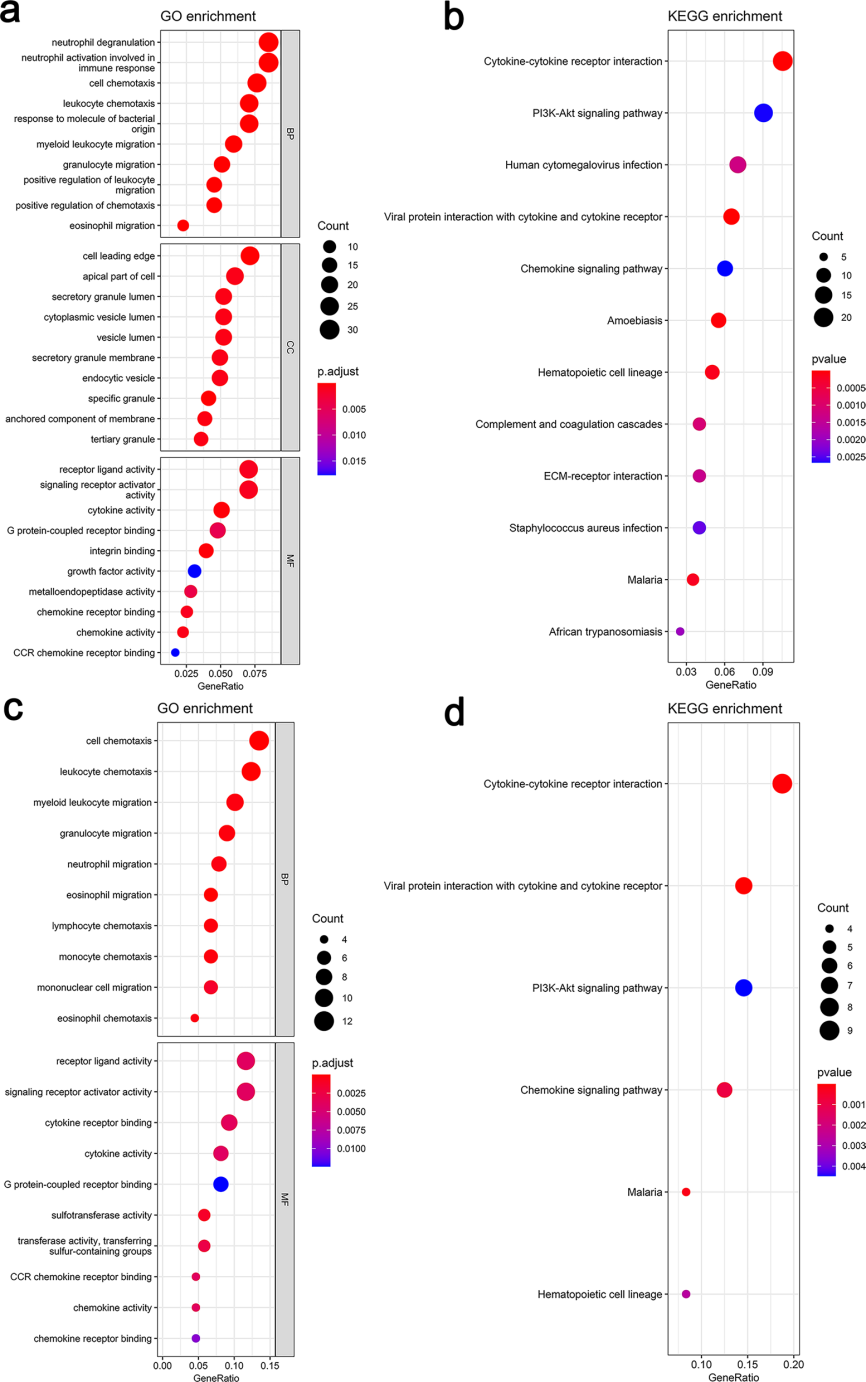
Significant GO terms and KEGG pathway analysis. **a** The top 10 significant terms for biological processes (BP), cellular component (CC), molecular function (MF) respectively in the Freiburg cohort. **b** The significant terms for KEGG pathways in the Freiburg cohort. **c** The top 10 significant terms for biological processes (BP), molecular function (MF) respectively in the Siena validation cohort. **d** The significant terms for KEGG pathways in the Siena validation cohort

In order to reveal the underlying biological functions and pathways that were correlated with the risk score, GO enrichment and KEGG pathway analyses were used to perform the DEGs between the high-risk and low-risk groups in the Freiburg and the Siena cohort. DEGs were enriched in inflammation- and immune-related GO and KEGG pathways such as receptor ligand activity, signaling receptor activator activity, cytokine activity, G protein-coupled receptor binding, CCR chemokine receptor binding, cytokine-cytokine receptor interaction, and chemokine receptor binding (Fig. 7) etc. As to Leuven cohort, the number of differential genes obtained under the same criteria was too small to perform functional enrichment analysis, so it was excluded.

**Fig. 8:**
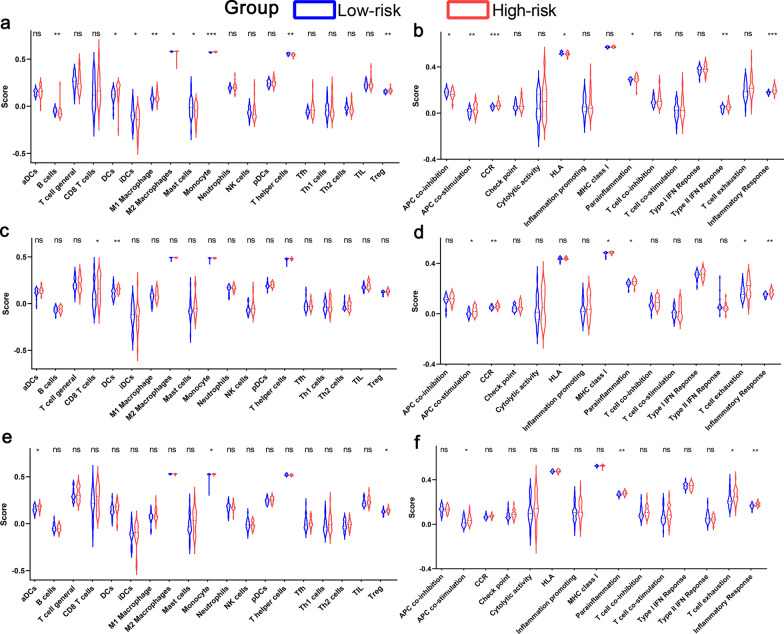
Comparison of the ssGSEA scores between low-risk and high-risk groups in the Freiburg cohort (**a, b**), in the Siena validation cohort (**c, d**), and in the Leuven validation cohort (**e, f**). The scores of 19 immune cells (**a, c, e**) and 15 immune-related functions (**b, d, f**) are displayed in violin plots. DC, Dendritic Cell; TIL, Tumor infiltrates lymphocytes; CCR, cytokine-cytokine receptor. P values were showed as: ns, not significant; *, P < 0.05; **, P < 0.01; ***, P < 0.001

To further explore the potential association between immune status and the risk score, ssGSEA was applied to quantify the enrichment scores of diverse immune cell subpopulations, related functions or pathways in the three cohorts. The scores of APC (antigen presenting cell) co-stimulation, parainflammation and inflammatory response were significantly higher in the patients of high-risk group compared with patients of low-risk group in the three cohorts (Fig. 8).

### The signature for the diagnosis of IPF

Compared with controls, risk score was significantly higher in the patients with IPF (P < 0.001, Additional file 6: Figure S6a). Based on the ROC curve analysis, the optimal cut-off value (9.148) of risk factor might have potential values in helping clinicians to identify patients with IPF, with a sensitivity of 90.3% and a specificity of 85.0% (AUC: 0.925, 95% CI 0.861–0.989; P < 0.001, Additional file 6: Figure S6b). According to ssGSEA, the dysfunction of immune response may be observed in the patients with IPF (Additional file 6: Figure S6c, d).

## Discussion

IPF is a chronic and serious lung disease. Lung tissue-based molecular genomic models [29, 30] have been found to be correlated with prognosis in patients with IPF. However, the procedure of lung biopsy is dangerous, which limits the applicability of such genomic signatures. The BAL cells gather on the outer surface of the alveoli and are usually collected by bronchoscope, and their gene expression is predictive of survival in IPF [9]. Therefore, the construction of the multi-gene signature of BAL cells is very important for the prediction of prognosis in patients with IPF. In this study, 51 ferroptosis-related DEGs were identified in IPF samples compared to normal controls in the Freiburg cohort from GSE70866 dataset, and 19 DEGs of them were associated with poor prognosis of IPF. We constructed a novel prognostic model integrating 8 ferroptosis-related DEGs and validated it in two external cohorts. In addition, the ROC curve confirmed the predictive value of risk score for prognosis. Functional analyses showed that inflammation- and immune-related pathways were enriched. Furthermore, according to DCA, using risk score or combination of risk score and GAP got more benefit compared with GAP only, suggesting that the 8-ferroptosis-related genes model can improve making clinical decisions to some extent.

Although several previous studies [15, 31] have suggested that a few genes might regulate ferroptosis in IPF, their relevance to survival in IPF patients is still largely unknown. Oxidative stress is thought to be involved in the development of alveolar damage, inflammation and fibrosis [32]. It has been found that in patients with IPF, lipid peroxidation and DNA oxidation are increased while antioxidant markers such as glutathione and Haem oxygenase (HO)-1 are reduced [12, 17]. We speculated that an imbalance of oxidants and antioxidants in the organism leads to increased production of ROS and altered iron homeostasis, which triggers ferroptosis characterized by iron-accumulation and participates in the progress of IPF. In this study, the 8-ferroptosis-related genes signature was associated not only with the diagnosis but also with the prognosis of IPF, which suggested that ferroptosis might participate the development and progression of IPF.

The prognostic model proposed in this study was consisted of 8 ferroptosis-related genes (NRAS, EMP1, SLC40A1, MYC, ANGPTL4, PRKCA, MUC1, GABARAPL1). In the current study, seven of the genes (NRAS, EMP1, SLC40A1, MYC, ANGPTL4, PRKCA, MUC1) in the prognostic model were up-regulated, while GABARAPL1 was down-regulated. Furthermore, these genes could be roughly divided into four categories, including inflammation or immune response (NRAS, PRKCA, MYC, SLC40A1), protein binding (GABARAPL1, EMP1), p53 binding (MUC1), angiogenesis (ANGPTL4) according to DAVID (http://david.abcc.ncifcrf.gov/) [33]. In terms of inflammation or immune response, the activation of PRKCA is involved in a calcium-mediated process participating in cystic fibrosis [34]. NRAS encodes GTPases involved in cell growth, proliferation and differentiation, and its protein products lead to downstream signaling events [35, 36]. MYC may promote the exacerbation of pulmonary fibrosis according to immune regulation [37, 38], and be a key gene according to the interaction network. MYC produces c-myc proto-oncoprotein, which acts down-stream of multiple growth factor signaling pathways, and MYC amplification was significantly associated with squamous cell lung carcinoma (SCC) in IPF patients [38]. SLC40A1 encodes Ferroportin which has an important role in the hypoferremic response to inflammation [39, 40]. P53 was considered to a novel regulatory factor in the process of ferroptosis with the dual effects on ferroptosis through transcriptional or post-translational mechanism [15, 41]. MUC1 may participate in the ferroptosis regulation by influencing p53 expression. ANGPTL4 (an angiopoietin-like protein belonging to a superfamily of secreted proteins) is involved in angiogenesis, which could be related to the development of pulmonary fibrosis [42]. A study [43] found that EMP1 could mediate cell density-regulated ferroptosis. GABARAP family members are involved in autophagosome maturation, and compared to normal tissues, the reduced expression of GABARAPL1 has been reported in various cancer cell lines [44]. The link between these results and ours remains to be determined, which need further study for the verification.

Although the underlying mechanisms of pulmonary diseases susceptibility to ferroptosis have been an intense research area in the past few years, the potential regulatory mechanism between IPF immunity and ferroptosis is still elusive. Based on the DEGs between different risk groups, functional enrichment analysis was performed, and we discovered that the inflammation- and immune-related pathways were enriched. Actually, inflammation- and immune-related pathways play vital role in the development of IPF, and immune processes can coordinate existing fibrotic responses [30, 45]. Interestingly, in this study, there was a significant difference in the process of antigen presentation between the low-risk group and the high-risk group. One possible theory is that ferroptosis cells emit different signals to attract antigen-presenting cells (APCs) to the site where cells die of ferroptosis [46]. Importantly, the pathogenesis of IPF is accompanied by a mass proliferation of macrophages. By changing phenotypes, such as classic activated macrophages (M1) and alternative activated macrophages (M2), macrophages participate in the pathogenesis of IPF and maintain the homeostasis of the lung environment [47]. In the Freiburg cohort, significant differences in M1 macrophages and M2 macrophages were observed between the low-risk group and high-risk group, however, there were no significant difference in the Leuven cohort and Siena cohort, which may be caused by the differences of patients or groups. In addition, higher risk scores were associated with impaired immune function, including the activity of the type II IFN response and T cell exhaustion as well as the fractions of Treg cells. Therefore, weakened immunity of high-risk patients may be a reason for their poor prognosis.

There are some limitations in this study. First, the model was constructed and validated based on the retrospective data from GEO, and the samples of every cohort were relatively small. A larger-sample prospective studies are needed to test its clinical application. Second, the inherent disadvantage of building a prediction model by considering only a single feature is inevitable. Many important prognostic genes in IPF might have been excluded. Third, clinical parameters such as lung function, treatment measures, underlying diseases and so on were absent in the datasets, thereby, the represent mean of the signature was limited. Forth, the treatment of patients with IPF was unknown in the three cohorts, which may affect outcome. Finally, it should be stressed that the relations between the risk score and immune activity have not yet been addressed in experiments.

## Conclusion

In conclusion, this study established a novel prognostic signature of 8 ferroptosis-related genes. The risk score might be an effective model to predict the poor prognosis of IPF. In addition, the potential mechanisms between prognosis and inflammation- and immune-related response in IPF remain poorly understood, which needs further investigation.

## Supplementary Information

**Additional file 1: Figure S1**. Construction of an 8-gene signature model in the Freiburg cohort. (a) LASSO coefficient profiles of the expression of 19 prognostic DEGs. (b) Selection of the penalty parameter (λ) in the LASSO model via 10-fold cross-validation.

**Additional file 2: Figure S2**. Survival analyses based on the optimal cut-off expression value of each gene in the Freiburg cohort. (a) NRAS. (b) EMP1. (c)SLC40A1. (d) MYC. (e) ANGPTL4. (f) PRKCA. (g) MUC1. (h) GABARAPL1.

**Additional file 3: Figure S3**. DCA for the survival prediction model of IPF in the Freiburg cohorts (a), Siena cohort (b), and Leuven cohort (c).

**Additional file 4: Figure S4**. Survival analyses based on the optimal cut-off expression value of each gene in the Siena validation cohort. (a) NRAS. (b) EMP1. (c)SLC40A1. (d) MYC. (e) ANGPTL4. (f) PRKCA. (g) MUC1. (h) GABARAPL1.

**Additional file 5: Figure S5**. Survival analyses based on the optimal cut-off expression value of each gene in the Leuven validation cohort. (a) NRAS. (b) EMP1. (c)SLC40A1. (d) MYC. (e) ANGPTL4. (f) PRKCA. (g) MUC1. (h) GABARAPL1.

**Additional file 6: Figure S6**. Comparison of the risk score between normal people and patients with IPF in the Freiburg cohort (a). Receiver operating characteristic (ROC) curve of the risk score for predictive value of IPF (b). Comparison of the ssGSEA scores between normal people and IPF patients in the Freiburg cohort (c, d). DC, Dendritic Cell; TIL, Tumor infiltrates lymphocytes; CCR, cytokine-cytokine receptor. P values were showed as: ns, not significant; *, P< 0.05; **, P< 0.01; ***, P< 0.001.

**Additional file 7: Table S1**. The 293 ferroptosis-related genes used in the study.

**Additional file 8: Table S2**. The annotated gene set file used in ssGSEA.

## Data Availability

The datasets used and/or analyzed in the current study are available in the GEO repository, https://doi.org/10.1164/rccm.201712-2551OC [9].

## Abbreviations

IPF: Idiopathic pulmonary fibrosis
DEGs: Differentially expressed genes
LASSO: Least absolute shrinkage and selection operator
ROC: Receiver operating characteristic
GEO: Gene Expression Omnibus
PCA: Principal component analysis
RCD: Regulated cell death
ROS: Reactive oxygen species
t-SNE: T-distributed stochastic neighbor embedding
DCA: Decision curve analysis
GO: Gene ontology
KEGG: Kyoto Encyclopedia of Genes and Genomes
SD: Standard deviation
IQR: Interquartile range
HR: Hazard ratio
AUC: Area under the curves
MF: Molecular function
BP: Biological processes
CC: Cellular component
BAL: Bronchoalveolar lavage
