# Supplementary figures and images for: An 8-ferroptosis-related genes signature from Bronchoalveolar Lavage Fluid for prognosis in patients with idiopathic pulmonary fibrosis
Source: BMC Pulm Med. 2022 Jan 5;22:15. doi: 10.1186/s12890-021-01799-7 (PMC8728942; doi:10.1186/s12890-021-01799-7)

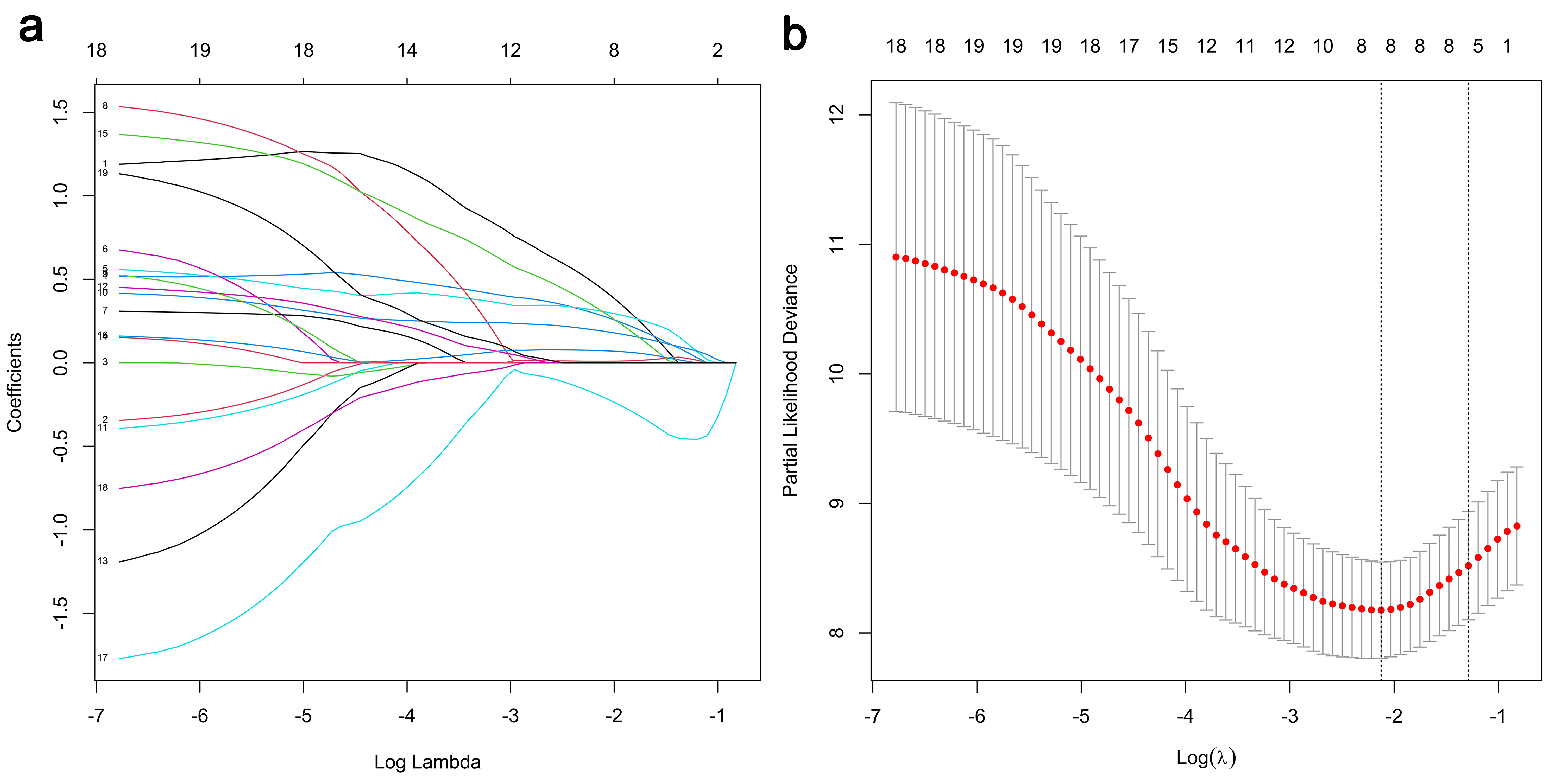

Supplement: Supplementary file 1 — Additional file 1: Figure S1. Construction of an 8-gene signature model in the Freiburg cohort. (a) LASSO coefficient profiles of the expression of 19 prognostic DEGs. (b) Selection of the penalty parameter (λ) in the LASSO model via 10-fold cross-validation. [file 12890_2021_1799_MOESM1_ESM.tif]

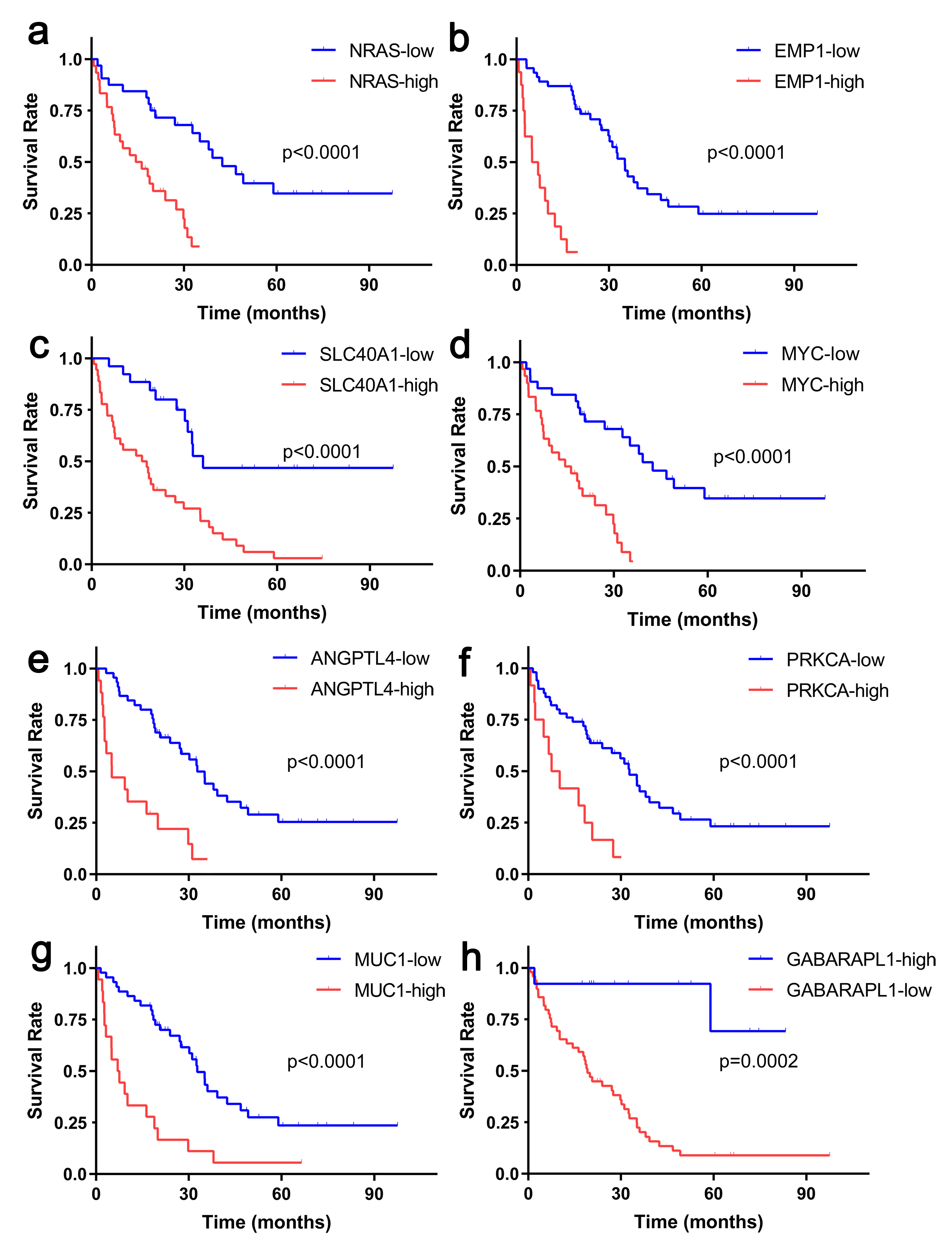

Supplement: Supplementary file 2 — Additional file 2: Figure S2. Survival analyses based on the optimal cut-off expression value of each gene in the Freiburg cohort. (a) NRAS. (b) EMP1. (c)SLC40A1. (d) MYC. (e) ANGPTL4. (f) PRKCA. (g) MUC1. (h) GABARAPL1. [file 12890_2021_1799_MOESM2_ESM.tif]

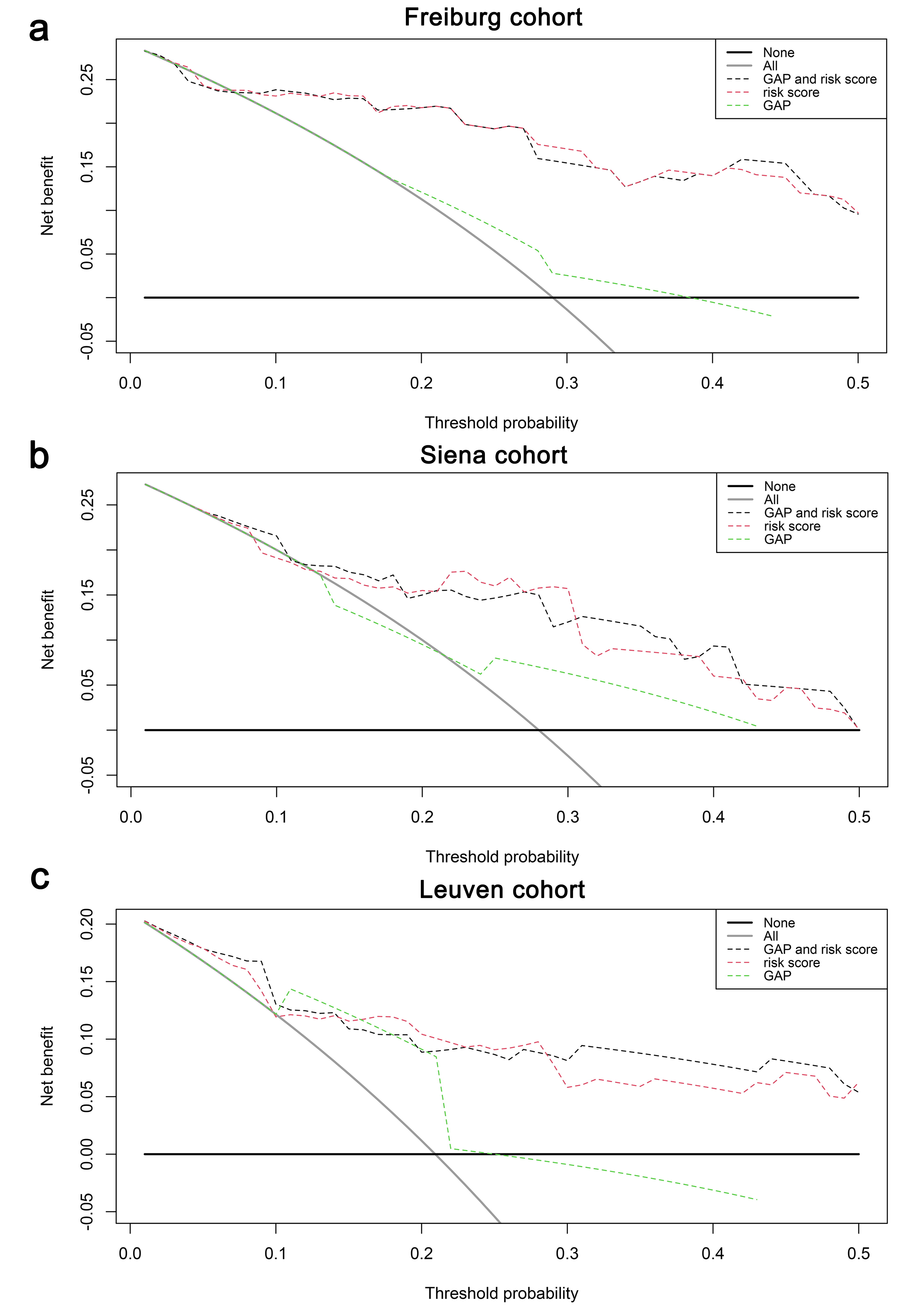

Supplement: Supplementary file 3 — Additional file 3: Figure S3. DCA for the survival prediction model of IPF in the Freiburg cohorts (a), Siena cohort (b), and Leuven cohort (c). [file 12890_2021_1799_MOESM3_ESM.tif]

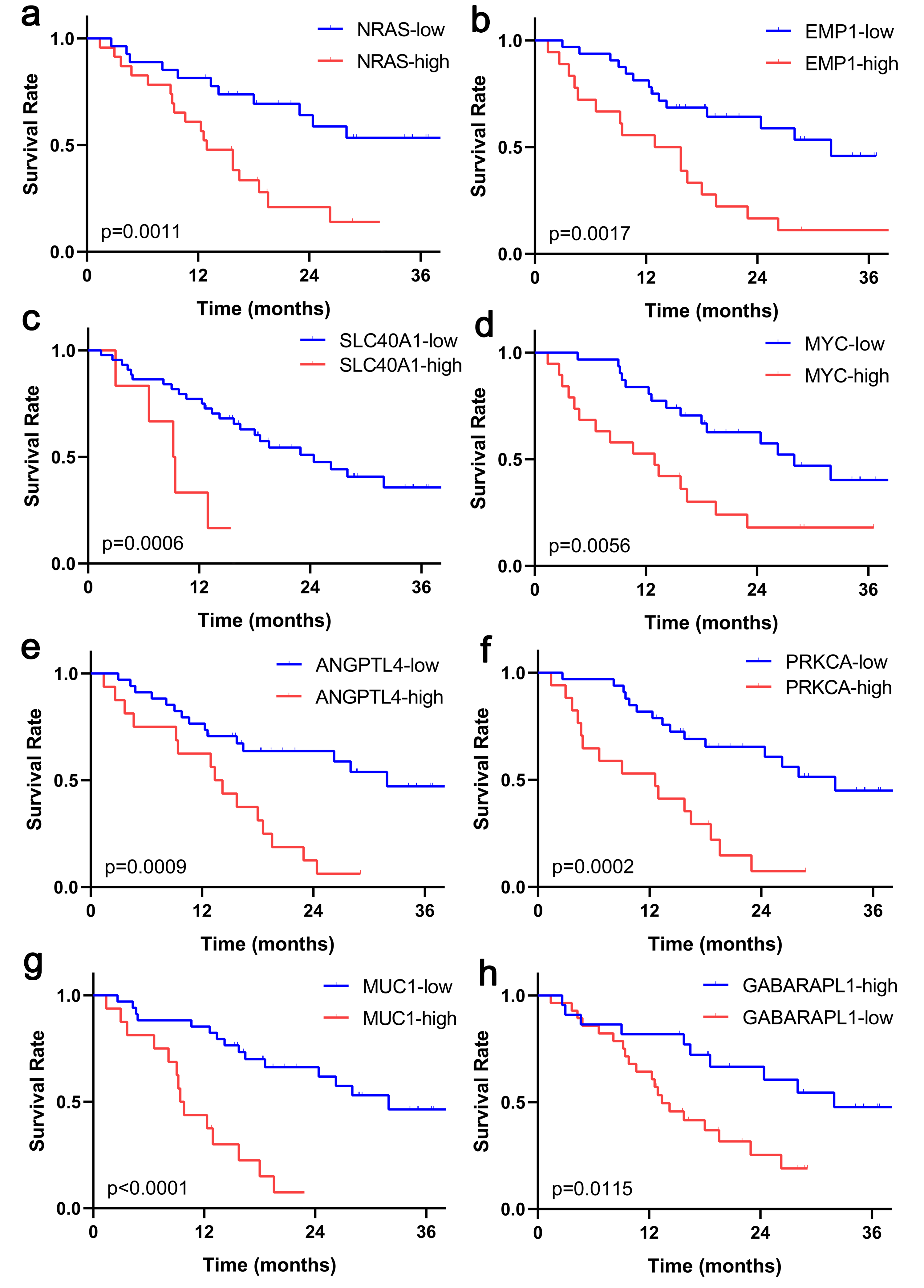

Supplement: Supplementary file 4 — Additional file 4: Figure S4. Survival analyses based on the optimal cut-off expression value of each gene in the Siena validation cohort. (a) NRAS. (b) EMP1. (c)SLC40A1. (d) MYC. (e) ANGPTL4. (f) PRKCA. (g) MUC1. (h) GABARAPL1. [file 12890_2021_1799_MOESM4_ESM.tif]

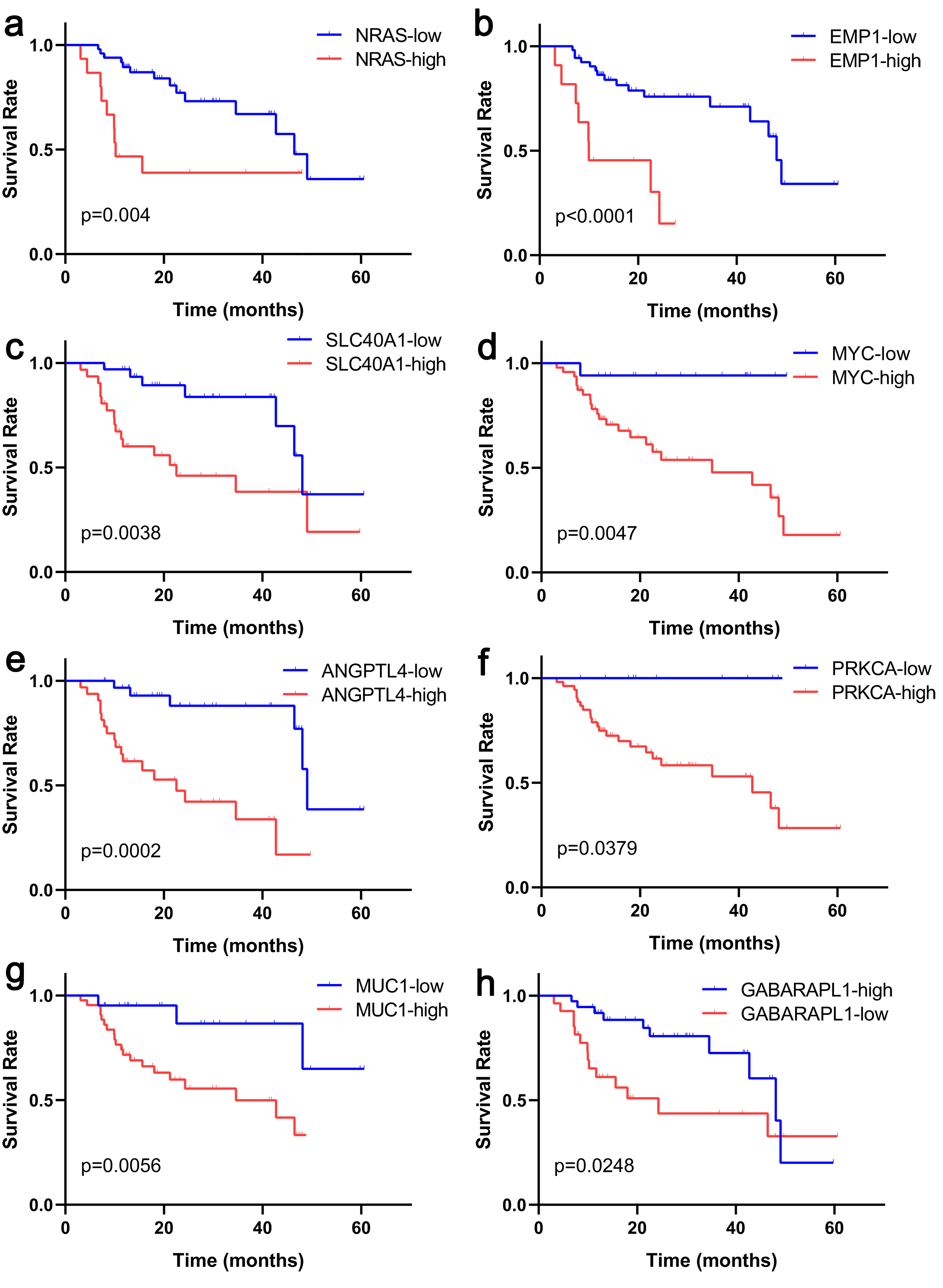

Supplement: Supplementary file 5 — Additional file 5: Figure S5. Survival analyses based on the optimal cut-off expression value of each gene in the Leuven validation cohort. (a) NRAS. (b) EMP1. (c)SLC40A1. (d) MYC. (e) ANGPTL4. (f) PRKCA. (g) MUC1. (h) GABARAPL1. [file 12890_2021_1799_MOESM5_ESM.tif]

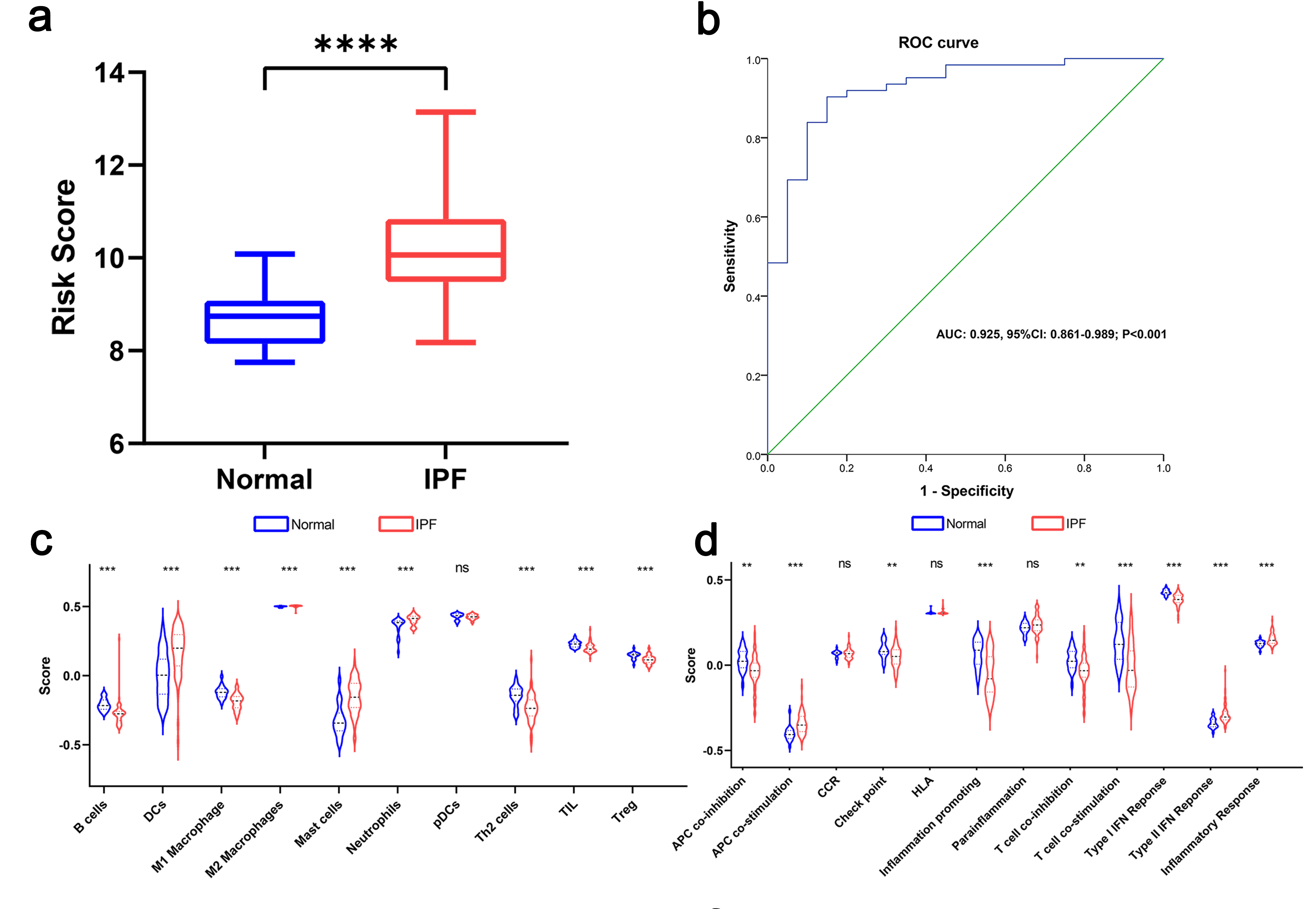

Supplement: Supplementary file 6 — Additional file 6: Figure S6. Comparison of the risk score between normal people and patients with IPF in the Freiburg cohort (a). Receiver operating characteristic (ROC) curve of the risk score for predictive value of IPF (b). Comparison of the ssGSEA scores between normal people and IPF patients in the Freiburg cohort (c, d). DC, Dendritic Cell; TIL, Tumor infiltrates lymphocytes; CCR, cytokine-cytokine receptor. P values were showed as: ns, not significant; *, P< 0.05; **, P< 0.01; ***, P< 0.001. [file 12890_2021_1799_MOESM6_ESM.tif]
